# The challenge of sustainability in healthcare systems: cost of radiotherapy in the last month of life in an Italian cancer center

**DOI:** 10.1007/s00520-020-05718-0

**Published:** 2020-09-28

**Authors:** Romina Rossi, Valentina Danesi, Ilaria Massa, William Balzi, Antonino Romeo, Flavia Foca, Oriana Nanni, Marco Maltoni, Mattia Altini

**Affiliations:** 1 Palliative Care Unit, Istituto Scientifico Romagnolo per lo Studio e la Cura dei Tumori (IRST) IRCCS, via P. Maroncelli 40, Meldola, FC 47014 Italy; 2 Healthcare Administration, Istituto Scientifico Romagnolo per lo Studio e la Cura dei Tumori (IRST) IRCCS, Via P. Maroncelli 40, Meldola, FC 47014 Italy; 3 Radiotherapy Unit, Istituto Scientifico Romagnolo per lo Studio e la Cura dei Tumori (IRST) IRCCS, via P. Maroncelli 40, Meldola, FC 47014 Italy; 4 Unit of Biostatistics and Clinical Trials, Istituto Scientifico Romagnolo per lo Studio e la Cura dei Tumori (IRST) IRCCS, via P. Maroncelli 40, Meldola, FC 47014 Italy

**Keywords:** End of life, Radiotherapy, Healthcare sustainability, Healthcare costs

## Abstract

**Purpose:**

Cost evaluation is becoming mandatory to support healthcare sustainability and optimize the decision-making process. This topic is a challenge, especially for complex and rapidly evolving treatment modalities such as radiotherapy (RT). The aim of the present study was to investigate the cost of RT in the last month of life of patients in an Italian cancer center.

**Methods:**

This was a retrospective study on a cancer population (*N*= 160) who underwent RT or only an RT planning simulation in an end of life (EOL) setting. The cost of RT procedures performed on patients was collected according to treatment status, care setting, and RT technique used. Costs were valued according to the sum of reimbursements relating to all procedures performed and assessed from the perspective of the National Health System.

**Results:**

The total cost of RT in the last month of life was €244,774, with an average cost per patient of €1530. Around 7.7% and 30.3% of the total cost was associated with patients who never started RT or who discontinued RT, respectively, while the remaining 62.0% referred to patients who completed treatment. Costs associated with outpatient and inpatient settings represented 54.3% and 38.6% of the total cost, respectively. The higher average cost per patient for the never-started and discontinued groups was correlated with patients who had a poor ECOG Performance Status.

**Conclusion:**

Improved prognostic accuracy and a better integration between radiotherapy and palliative care units could be a key to a better use of resources and to a reduction in the cost of EOL RT.

## Introduction

The quality of care delivered to cancer patients near the end of life (EOL) has become a hot topic for researchers [1–14] and is contributing to the development of interventions and policies to guarantee appropriate EOL care and to optimize the use of healthcare resources [2, 6, 7, 10, 15, 16]. Little information is available on the correlation between EOL care and healthcare costs, the majority of economic evaluations having been conducted in the USA [1, 5, 11] whose healthcare system differs significantly from most of the European ones. However, all the studies agree that reducing EOL costs represents a constant challenge within the healthcare system [9, 10].

It is known that radiotherapy (RT) is a pivotal approach in cancer treatments, with around 50% of all cancer patients undergoing RT to manage their illness and 8–18% of these receiving treatment in the last 30 days of life [8, 11].

Given the advancements made in cancer care, including radiotherapy technique, the distinction between curative RT and palliative RT (PRT) has become blurred. The goal of therapy in patients with metastatic disease from solid tumors is generally palliative, but improved systemic therapies have, in certain circumstances, led to longer overall survival times [12].

There is still very little information in the literature on the use and cost of RT at the EOL, many of the studies not focusing on this specific RT setting [17–19], some limited to the analysis of a specific cancer site [14, 20–23] and others dealing with a specific RT technique [23–27]. There is also a paucity of methodological detail in published studies, along with considerable variation in the calculated cost estimates.

This heterogeneity of methods in cost evaluation clearly compromises benchmarking within the context of RT [28, 29]. The generation of high-quality cost data, especially for complex and rapidly evolving treatment modalities such as those used for RT, is needed to guarantee the sustainability of the healthcare system because these novel RT techniques are often associated with high costs [28].

Given the above premises, we carried out the present retrospective study at our institute (Istituto Scientifico Romagnolo per lo Studio e la Cura dei Tumori (IRST) IRCCS) to study RT costs in the last 30 days of life of a population of cancer patients. This analysis is a part of the beginning of our process to develop new models and tools in order to optimize use of healthcare resources in PRT.

Costs were evaluated on the basis of treatment status, care setting, and RT technique used.

## Materials and methods

This retrospective study is a secondary cost analysis conducted on a patient population included in a previous study [30].

Of the 2444 patients referred to our institute for RT and who died between January 01, 2009, and December 31, 2015, 160 (6.5%) underwent RT the last 30 days of life, both as a new treatment or continuing a course started more than 30 days before they died. We included also patients that received only RT planning simulation in the last 30 days of life [30]. As in the previous study, this population was divided into 3 main subgroups on the basis of treatment status at the time of death:

1. Never-started: patients considered for RT who only underwent RT planning procedures such as a CT scan but did not start treatment;
2. Discontinued: patients who started RT but prematurely discontinued therapy;
3. Completed: patients who completed the planned RT;

Twenty (12.5%) patients were included in the never-started group and 49 (30.6%) in the discontinued group due to worsening medical conditions or death. A total of 91 (56.9%) patients who completed treatment were included in the last group. Patient characteristics, RT techniques, and fraction schedules are shown in Table 1.

**Table 1:** Patient characteristics, radiotherapy (RT) technique, and fraction schedule according to treatment status at EOL (within 30 days of death)*

		RT treatment status		
	Total population (%) *N* = 160	Never-started (%) *N* = 20 (12.5%)	Discontinued (%) *N* = 49 (30.6%)	Completed (%) *N* = 91 (56.9%)
Median age at death, years (range)	67 (25–90)	71 (51–89)	68 (35–87)	66 (25–90)
Gender				
Male	100 (62.5)	11 (55.0)	30 (61.2)	59 (64.8)
Female	60 (37.5)	9 (45.0)	19 (38.8)	32 (35.2)
ECOG PS				
0–1	73 (45.6)	8(40.0)	23 (47.0)	42 (46.1)
2	44 (27.5)	7 (35.0)	13 (26.5)	24 (26.4)
3–4	42 (26.3)	5 (25.0)	13 (26.5)	24(26.4)
Undocumented	1 (0.6)	0 (0.0)	0 (0.0)	1 (1.1)
Primary cancer site				
Lung	62 (38.8)	1 (5.0)	14 (28.6)	39 (42.8)
Gastrointestinal tract	24 (15.0)	0 (0.0)	10 (20.4)	11 (12.1)
Urological tract	14 (8.8)	2 (10.0)	7 (14.3)	6 (6.6)
Breast	12 (7.5)	0 (0.0)	4 (8.1)	8 (8.8)
Head and neck	12 (7.5)	1 (5.0)	6 (12.2)	5 (5.5)
Other sites^1^	36 (22.4)	7 (20.0)	8 (16.3)	22 (24.2)
RT technique^2^				
Standard technique	95 (67.9)	–	36 (73.5)	59 (64.8)
High-precision technique	45 (32.1)	–	13 (26.5)	32 (35.2)
Fraction schedule per treatment course				
1	16 (11.4)	–	-	16 (17.6)
2–9	61 (43.6)	–	19 (38.8)	42 (46.1)
10	40 (28.6)	–	18 (36.7)	22 (24.2)
˃ 10	23 (16.4)	–	12(24.5)	11 (12.1)

^1^Other sites included hepatobiliary system, skin, female reproductive system, hematopoietic and lymphoid tissue, brain, soft tissue, and testes

^2^RT technique and fraction scheduled are reported for completed and interrupted groups (*N* = 140)

*More details about patient’s characteristics can be found in [30]

The cost and amount of resources used in the 30 days before death were valued on the basis of the RT technique used. Information on procedures performed outside the 30-day window was not collected for 34 (21.3%) patients who started RT more than 30 days before they died.

RT techniques were identified on the basis of the complexity of each procedure, as follows:

- Standard technique: 2-dimensional (2D) including cobalt therapy (up to 2013), 3-dimensional conformal (3D) RT, and volumetric modulated arc therapy (V-MAT) technique
- High-precision technique: TomoTherapy (used at our institute since 2013)

In the present analysis, costs were valued as the sum of reimbursements for the procedures performed as part of the RT care pathway, including first visit, planning CT scan, medical physics treatment planning, and treatment sessions on the basis of the number of fractions delivered. Costs were assessed from the perspective of the Italian National Health System. The Regional Healthcare Range of Fees table [31] was used to determine the unit cost of each RT procedure per patient in an outpatient setting. With regard to the inpatient setting, we set up a methodology to calculate the cost of RT procedures. Rather than computing the entire DRG (diagnosis-related group)–related costs, we identified each RT procedure reported in the Hospital Discharge Cards of patients and summed up costs according to the Regional Healthcare Range of Outpatient Fees table to obtain the total cost for inpatients.

We retrieved the Eastern Cooperative Oncology Group (ECOG) Performance Status (PS) scores of patients on the day of the RT consultation or up to 7 days before from electronic health records. The average cost per patient was estimated for each subgroup on the basis of ECOG PS scores (good [0], moderate [2], and poor [3, 4]).

### Data sources

Patients were identified through IRST electronic medical records (CCE Log80 2.6 of Log80 S.r.l) in which the date of death was registered. Patient data were cross-checked with those of IRST’s Radiation Oncology database (MOSAIQ version 2.64) and with an internal administration software system used to collect and manage patient reimbursements at our institute (Cdg2007 of CEDAF S.r.l).

### Statistical analysis

Continuous data were expressed as median (range) while categorical data were expressed as numbers and percentages. Costs were expressed as total costs and as average cost per patient. Bar graphs were used to better explain data. Due to descriptive nature of this work, statistical comparisons among subgroups were not done.

## Results

**Fig. 1:**
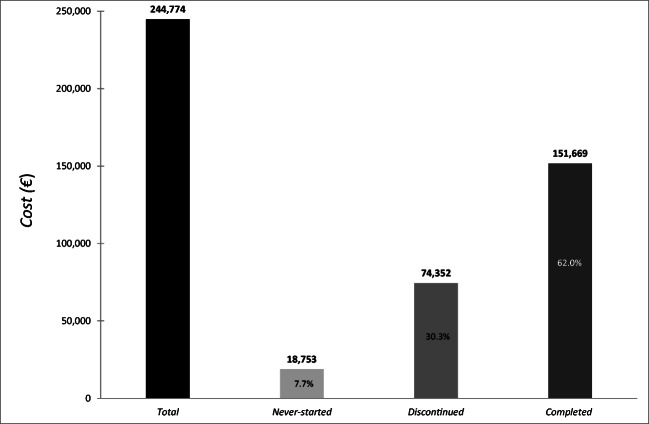
Cost of radiotherapy (RT) in the last 30 days of life of advanced cancer patients. The black bar shows the total cost for the entire patient population, while the other bars represent RT costs according to patient treatment status (never-started, discontinued, and completed)

**Fig. 2:**
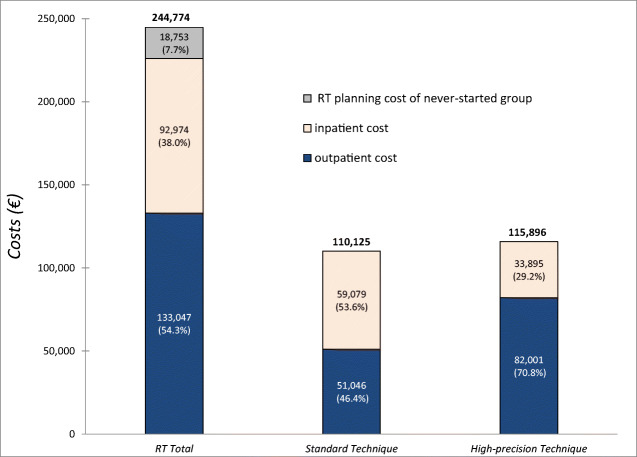
The bar on the left shows the total cost of RT for the entire patient population subdivided into the RT planning cost of the never-started group and inpatient and outpatient setting costs. The middle bar shows the RT cost for the standard technique subdivided into inpatient and outpatient setting costs, and the right bar shows the cost of RT for the high-precision technique subdivided into inpatient and outpatient setting costs

The overall cost of RT delivered to the study population (*N* = 160) in the last 30 days of life during the study period (between 2009 and 2015) was €244,774. Around 7.7% of the overall cost was attributed to the never-started group and 30.3% to the discontinued group, while the remaining 62.0% was associated with the patients who completed treatment (Fig. 1). The average total cost per patient was €1,530. Costs associated with outpatient and inpatient settings were 54.3% and 38.0%, respectively (Fig. 2). The costs of standard and high-precision RT techniques were almost identical (Fig. 2), although the number of procedures delivered was significantly different (*N* = 1548 for standard technique vs. *N* = 849 for high-precision technique). The average cost per patient for standard technique was €1,159, while much more was the average cost €2,575 associated with high-precision RT technique. With regard to the standard technique, outpatient and inpatient costs were comparable as the number of procedures delivered was almost the same in either setting (*N* = 782 for outpatients vs. *N*= 766 for inpatients). Conversely, the high-precision technique was associated with a higher cost in the outpatient setting, where it was more frequently used (*N* = 603 for outpatients vs. *N* = 246 for inpatients).

**Fig. 3:**
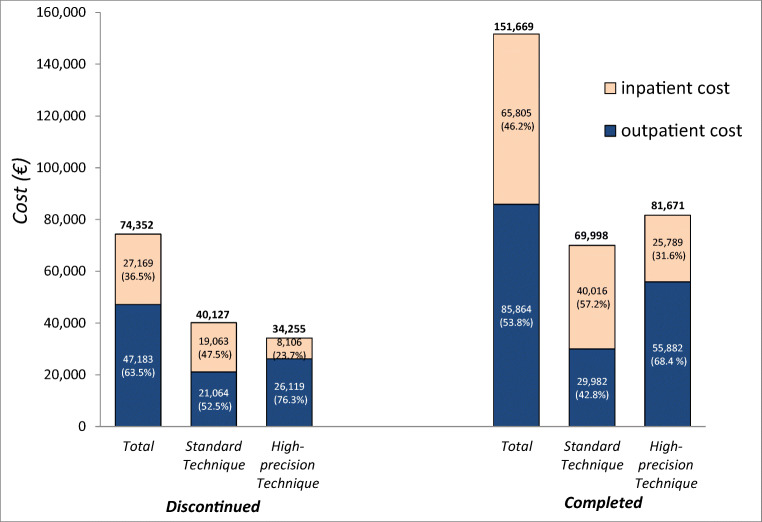
Cost of RT in discontinued and completed groups subdivided according to RT technique and in/outpatient setting. The left bar of each group shows the total cost while the middle and right bars show cost distribution according to treatment technique subdivided by inpatient and outpatient setting

The overall cost of RT in the discontinued group was €74,352 (Fig. 3), with an average cost per patient of €1,571. A large percentage of this cost (63.5%) was for outpatient procedures. The cost of the standard technique was €40,127, while that of the high-precision technique was €34,225, accounting for 54.0% and 46.0% of the total cost, respectively. The outpatient expenditure was the highest, accounting for 52.5% of the standard technique cost and 76.3% of the high-precision technique cost (Fig. 3).

The overall cost of the group that completed treatment was €151,669 (Fig. 3), with an average cost per patient of €1,667. The highest cost (53.8%) was associated with outpatient setting. The cost of the standard techniques was €69,998, while that of the high-precision technique was €81,671 (46.2% and 53.8% of the total cost, respectively). A comparison between techniques revealed an opposite pattern, the highest cost for inpatients being the standard technique (57.2%) while that for outpatients was the high-precision technique (68.4%) (Fig. 3).

With regard to the use of resources, the number of outpatient procedures (*N* = 520) was considerably higher than that of inpatient procedures (*N* = 307) for the discontinued group, whereas the difference was less marked for the group that completed treatment (*N* = 865 for outpatients, *N* = 705 for inpatients). For the high-precision technique, the number of outpatient procedures was 2- and 4-fold higher than that observed in the inpatient setting for the discontinued and completed groups, respectively.

**Fig. 4:**
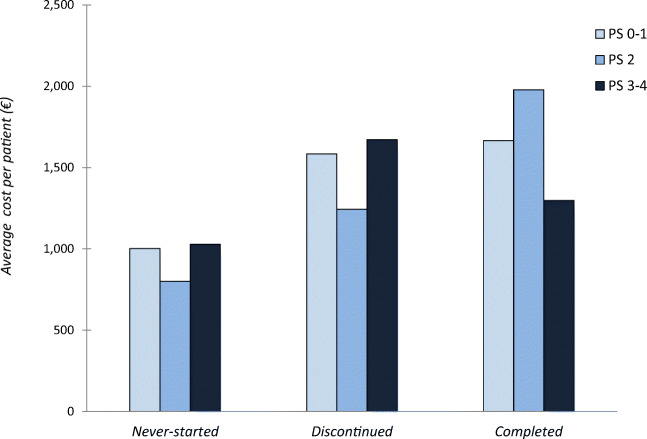
Average cost per patient in the never-started, discontinued, and completed groups according to good (0–1), moderate (2), and poor (3–4) ECOG PS

A higher average cost per patient was associated with poor ECOG PS (3–4) in never-started and discontinued patients and with ECOG PS 1–2 in the completed group (Fig. 4). One patient in the completed group was excluded because of undocumented PS in the electronic medical record (Table 1).

## Discussion

The aim of this retrospective study was to investigate the cost of RT during the last 30 days of life in a population of cancer patients. There are very few data in the literature on the cost of EOL RT, even though the European radiotherapy community agrees that such information is crucial for optimizing access to RT services and for reimbursement purposes [28]. To the best of our knowledge, ours is the first study carried out in an Italian institution to evaluate the cost of an EOL RT service. Our results showed that, out of almost €250,000 spent on RT in the last 30 days of life, 30.3% was used for the discontinued group who obviously did not benefit from treatment and 7.7% was used for never-started patients. There is clearly ample leeway for improving the use of RT resources. Although short-fractionation or single-dose RT has a place in the treatment of EOL patients, there is still a real risk of overusing RT in this setting.

As expected, when outpatient and inpatient settings were compared in our study, costs were higher for the former and associated with high-precision techniques. This was probably due to the fact that high-precision RT was more often reserved for patients who would have better compliance with the treatment than those in poorer conditions or who received combined treatments such as radio-chemotherapy.

Although the tendency to treat patients up to the very end and refer them for palliative care when it is too late is widely acknowledged as an indicator of poor-quality care [7], this practice is still all too common. Our findings corroborate those of previous studies on the trend in aggressiveness of cancer care near the end of life, highlighting a fairly high intensity of care [2, 5] and inappropriately lengthy treatment regimens [32]. Thus, if “inappropriate treatment” is defined as “interventions that are ineffective in achieving the desired goals, or are a disservice to patients who are subjected to ongoing and likely uncomfortable conditions with no direct benefit” [33], all costs pertaining to the never-started and discontinued groups can be considered inappropriate costs, regardless of the treatment intent, Figs. 1 and 3 (which provides greater detail for the never-started and discontinued groups) show the amount of resources used for non-beneficial treatments in our population, suggesting that careful patient selection and more accurate survival prognostication are key to reducing the risk of inappropriate therapies and costs [34].

Concerning ECOG PS, our study indicates that the higher average cost per patient for never-started and discontinued groups was associated with patients with poor PS. As suggested in other studies [35, 36], ECOG PS could be a crucial factor for estimating life expectancy in the last month of life, gauging treatment appropriateness, and reducing improper costs, especially in a palliative care setting.

There are several limitations to this paper, the main ones being its retrospective design and the fact that the data were collected over a long period of time, with suboptimal completeness. Furthermore, the monocentric design of the study does not express the reality of all Italian cancer centers. Secondly, out-of-pocket costs for patients and indirect costs such as those for staff, equipment, and maintenance were not taken into account. Moreover, our study did not collect detailed data on the palliative versus curative intent of therapy, making it more difficult to determine whether treatment was clinically appropriate. Differences in costing methodologies, assessment timeframes [17–19], tumor sites [14, 20, 23], and healthcare system may help to explain the observed variations in computed costs, rendering a comparison across studies complicated [28].

We wish that more Italian cancer centers will carry out this kind of analysis and share data in order to do benchmarking for the optimization of healthcare system resources within the Italian context of RT. The main strength of the present study is that the costing methodology was based on the extraction of real data from the institute’s RT database and an internal administration software system, grouping costs into the 2 main RT techniques and settings.

For the first time, costs were evaluated according to RT treatment status (never-started/discontinued/completed), which showed more clearly the costs that could be re-allocated to achieve better results for patients. From a clinical point of view, it is important to avoid aggressiveness of care at EOL, choose appropriate treatment schedules and RT techniques according to the patient’s life expectancy, and establish the correct timing of RT to provide relief from or prevent patient symptoms [36].

In conclusion, the above findings have enabled us to reorganize the RT Unit of our institute. In fact, since 2016, we have been using a new integrated approach between our Radiotherapy and Palliative Care Units to optimize the use of healthcare resources and guarantee appropriate EOL care for advanced cancer patients. This model is also backed by substantial evidence that palliative care combined with standard cancer care improves patient and caregiver outcomes in terms of quality of life (QoL), survival, use of healthcare services, and costs [37–39]. Into this integrated approach, the systematic analysis of all data collected will hopefully provide the answer to the many open questions remaining in this challenging healthcare area.

## Data Availability

The datasets generated and/or analyzed during the current study are available from the corresponding author on reasonable request.
